# Great inclination to smoke among younger adults coming from low-socioeconomic class in Thailand

**DOI:** 10.1186/1755-7682-4-29

**Published:** 2011-08-28

**Authors:** Sunsanee Mekrungrongwong, Keiko Nakamura, Masashi Kizuki, Ayako Morita, Tewarit Somkotra, Kaoruko Seino, Takehito Takano

**Affiliations:** 1 International Health, Division of Public Health, Graduate School of Medical and Dental Sciences, Tokyo Medical and Dental University, Tokyo, Japan; 2 Health Promotion, Division of Public Health, Graduate School of Medical and Dental Sciences, Tokyo Medical and Dental University, Tokyo, Japan; 3 Department of Community Dentistry, Faculty of Dentistry, Chulalongkorn University, Bangkok, Thailand

**Keywords:** Social gradient in health, health promotion, inequity in health, socioeconomic factors, smoking habit, Thailand

## Abstract

**Background:**

WHO estimates that 8.4 million deaths will be counted a year due to tobacco by 2020, and 70% of those deaths will occur in developing countries. Examination of the magnitude of socioeconomic differences in smoking between different age groups reveals specific groups anti-smoking programs should target on. This study aimed to measure socioeconomic gradients related inequality in smoking behavior among young and old Thai male population, where general progress in reduction on smoking prevalence has already shown.

**Methods:**

Data of Thai males aged 21 years and older from Health and Welfare Survey and Socio-Economic Survey, Thailand, 2006 were used in the analyses. Variables in education, household income, age, marital status, and region of residence were used to examine their associations with smoking status.

**Results:**

Of the 12,200 respondents, overall prevalence of smoking among males aged 21 years and older was 41.5%. Lower education was strongly associated with smoking (OR 3.15; 95% CI, 2.74-3.62). Youngest age, reside in South region and lowest income were more associated with smoking (OR = 2.66, 1.30, and 1.91, p < 0.05, respectively). Smoking among young adults (age 21-30) (OR = 5.88; 95% CI, 4.3-8.0) showed stronger gradients with educational level than that among older adults (OR = 3.96; 95% CI, 2.8-5.3).

**Conclusions:**

The inverse associations between smoking prevalence and socioeconomic status among the Thai adult male population were consistently confirmed. The social gradient in smoking was greater among young adult males than that among older adult males.

## Background

Smoking tobacco is a major public health problem. Tobacco kills 4 million every year. There has been a progress in smoking control at policy level; however, most countries are still struggling to control. By 2020, World Health Organization (WHO) estimates that the number of tobacco victims would rise to 8.4 million and 70% of them would occur in developing countries [1].

In addition to male gender, lower socioeconomic status has been revealed to closely link with smoking status in many developed countries and a few developing countries where data is available. Studies have consistently shown that cigarette smoking was more prevalent among lower educational class, lower occupational class, and residences of deprived areas [2]. Physical, social, and psychological environment of low socioeconomic class place them under a more vulnerable situation to cigarette smoking. For example, poor housing quality including crowdedness has shown to increase perceived stress and is associated with higher smoking consumption [3]. People with lower socioeconomic status in urban areas are particularly vulnerable [4-6]. Tobacco companies carry out active tobacco promotion in urban areas where they can approach to a large number of people, and price is more acceptable to residents in urban areas than from rural areas.

Although socioeconomic gradients in smoking has been observed across age groups, strength of the associations have shown to be not consistent between younger adults and older adults [7-11]. For example, studies conducted in 12 European countries [12] found that younger people with lower educational class were more likely to smoke than older people with lower educational class, compared with the same age groups with high education. However, a study in India [13] showed that cigarette consumptions were more prevalent among older people than younger people of rural residents.

Evidence of socioeconomic gradients in smoking has been gathered mainly from North America and Western Europe [13-18], and evidence from developing countries is limited. In order to develop effective smoking control intervention programs worldwide, we need to build more evidence from developing countries and understand which demographic groups of populations (i.e., age, socio economic status) require particular attention.

Thailand has ratified the Framework Convention on Tobacco Control since 2004. The country's first actions started with compulsory health warning signs on tobacco products back in 1974 [19]. Within ten years, NGOs started actively spearheading anti-smoking movement, and the National Committee for Control of Tobacco Use (NCCTU) was established in 1989. A number of major tobacco control laws were passed since then. Cigarette advertisement is banned in all media except live telecasts and imported magazines, and prohibited sales to minors (under 18) and vending machine sales. Health warning signs are printed in easily readable colors, and cover more than 50% of a package accompanied with graphic images since 2004. Smoking is prohibited in public buildings and workplace since 90s, and extended to pub, bar and market since 2008. Cigarette was taxed 69% since 1994 and the tax was increased to 79% in 2006. As a result of a number of smoking control policies, smoking prevalence in Thailand has progressively been declined over decades [20]. It was almost halved in men (from 65.2% to 37.2%) and more than halved in women (from 5.4% to 2.1%) in 2004, compared with 1981. Smoking prevalence in minors was also dramatically reduced between 2000 and 2004 (from 0.91% to 0.10% among 11-14 yrs-old boys and 15-19 yrs-old boys, from 27.36% to 11.2%). Still, 2 out of 3 adult men smoke across age and there are needs to identify which socioeconomic and demographic groups require prioritized policy efforts to reduce health inequity.

The present study examines an association between socioeconomic and smoking status, across age groups among adult men in Thailand. The association will be examined using national representative samples.

## Methods

### Data source

We used data of male subjects aged 21 years and above (n = 12,200) from the Health and Welfare Survey (HWS) 2006 and the Socio-Economic Survey (SES) 2006. HWS surveys health behaviors including smoking, and SES surveys socioeconomic status and demographics among HWS subjects. Both surveys were cross-sectional, conducted by the National Statistics Office of Thailand among 22,517 households selected by a two-stage stratification sampling with weight adjustment to sample a national representative.

### Measurements

Current smoking status, starting age, and average cigarette consumptions per day were obtained. Respondents were classified into one of the following five categories based on the answers: 1) non-smoker, 2) ex-smoker, 3) light smokers (= < 10 cigarettes/day), 4) moderate smokers (11-20 cigarettes/day), and 5) heavy smoker (> = 21 cigarettes/day).

Educational level and average household income were employed as indicators of socioeconomic status. Study subjects were classified into three levels of education based on total years of formal education: less than 6 years as low, 7 to 12 years as middle, and more than 13 years as high. They were also classified into five levels of income based on their average household income based on quintiles. The lowest quintile (1) indicates the poorest and the highest quintile (5) indicates the richest.

Measured demographic variables included age, marital status and residential regions. Respondents were classified into five age groups (21 to 30, 31 to 40, 41 to 50, 51 to 60, and 61 years above), three marital status (i.e., single, married and other which includes divorced, widowed, separate or unknown; and five regions (i.e., Bangkok, Central (exclude Bangkok), North, Northeast, and South areas).

### Statistical analysis

Prevalence of smoking status was calculated according to educational and income levels.

Odds ratio of smoking status was calculated according to educational and income level with a high educational group being a reference.

The logistic regression was performed to examine independent variable to show relationships with smoking status, with the high educational level group, high income quintile and older age group (age more than 61 years old) as the reference group. Age, education, region, marital status, and income per household quintile were included in the model as independent variables. Odds ratio and 95% confidence intervals were calculated from regression coefficients.

A slope index of inequality (SII) and a relative index of inequality (RII) of prevalence of smoking were estimated between the high and low educational level groups for individual age-groups. This age-group specific SII was estimated as a slope in a weighted linear regression model with the prevalence of smoking as a dependent variable and the mean percentile score of each age-group as an independent variable. Weights applied to a linear regression were calculated based on the numbers of data for each age-group. The RII was computed as the ratio of the SII to the age-group specific mean prevalence.

All the analyses above were carried out using SPSS version 15.

## Results

Table 1 shows smoking status and socioeconomic and demographic characteristics of study subjects. 41.5% of the subjects were currently smoking and 15.4% was not current smokers but used to be. Of the smokers, 2.3% were heavy smokers.

**Table 1:** General characteristics of the respondents aged 21 years and older, 2006 (N = 12,200)

Characteristics	Male	
	n	%
**Ages**		
21-30	1,702	**14.0**
31-40	2,678	**22.0**
41-50	3,054	**25.0**
51-60	2,297	**18.8**
61 and older	2,469	**20.2**
**Education**		
Lower Education	6,591	**56.4**
Middle Education	3,187	**27.3**
High Education	1,910	**16.3**
**Region**		
Bangkok	649	**5.3**
Central	3,548	**29.1**
North	3,281	**26.9**
Northeast	3,141	**25.7**
South	1,581	**13.0**
**Marital status**		
Single	1,589	**13.0**
Married	9,558	**78.3**
Other*	1,053	**8.6**
**Smoking Status**		
Non-smoker	5,257	**43.1**
Ex-smoker	1,877	**15.4**
Smoker < = 10 cigarettes/day	3,753	**30.8**
Smoker 11-20 cigarettes/day	1,198	**9.8**
Smoker > = 21 cigarettes/day	115	**0.9**
Total	12,200	**100.0**

* "Other" includes divorced, widowed, separated and unknown

Table 2 shows the age-adjusted prevalence of smoking status by socio-demographic status. About 1/3 were light smokers, 1/10 were medium smokers, and about 1% were heavy smokers among middle-aged and older men. Prevalence of smoking among the subjects with low education was double of the subjects with high education across all degrees of smoking. Furthermore, prevalence of ex-smoker status was higher among the subjects with low education than the subjects with middle or high education. Similarly, smoking prevalence declines proportionally as income increases. Prevalence of smoking among the subjects with the lowest income was almost double of the subjects with the highest income. With respect to residential region, there was a little difference. Currently smoking was more prevalent who reported other marital status (46.1%) than it was for those who were married 41.4% or single 39.4%.

**Table 2:** Smoking status in men age more than 21 years old by each socio-demographic factor (n = 12,200)

Socioeconomic		Smoking status									
Status	Total	Non smoker		Ex smoker		< = 10 cigarettes/d		11-20 cigarettes/d		> = 21 cigarettes/d	
	n	n	**%**	n	**%**	n	**%**	n	**%**	n	**%**
**Ages**											
21-30	1702	868	**51.0**	133	**7.8**	589	**34.6**	104	**6.1**	8	**0.5**
31-40	2678	1135	**42.4**	309	**11.5**	906	**33.8**	311	**11.6**	17	**0.6**
41-50	3054	1231	**40.3**	436	**14.3**	980	**32.1**	371	**12.1**	36	**1.2**
51-60	2297	976	**42.5**	391	**17.0**	648	**28.2**	250	**10.9**	32	**1.4**
61 and older	2469	1047	**42.4**	608	**24.6**	630	**25.5**	162	**6.6**	22	**0.9**
Education*											
Lower Education	6591	2374	**36.0**	1086	**16.5**	2302	**34.9**	753	**11.4**	76	**1.2**
Middle Education	3187	1454	**45.6**	463	**14.5**	941	**29.5**	305	**9.6**	24	**0.8**
High Education	1910	1249	**65.4**	246	**12.9**	305	**16.0**	100	**5.2**	10	**0.5**
Region*											
Bangkok	649	337	**51.9**	97	**14.9**	163	**25.1**	46	**7.1**	6	**0.9**
Central	3548	1755	**49.5**	415	**11.7**	968	**27.3**	371	**10.5**	39	**1.1**
North	3281	1292	**39.4**	701	**21.4**	993	**30.3**	271	**8.3**	24	**0.7**
Northeast	3141	1242	**39.5**	494	**15.7**	1071	**34.1**	304	**9.7**	30	**1.0**
South	1581	631	**39.9**	170	**10.8**	558	**35.3**	206	**13.0**	16	**1.0**
Marital status*											
Single	1589	821	**51.7**	141	**8.9**	506	**31.8**	111	**7.0**	10	**0.6**
Married	9558	4043	**42.3**	1561	**16.3**	2884	**30.2**	979	**10.2**	91	**1.0**
Other **	1053	393	**37.3**	175	**16.6**	363	**34.5**	108	**10.3**	14	**1.3**
Household income quintile*											
1 (lowest)	2440	795	**32.6**	359	**14.7**	1019	**41.8**	241	**9.9**	26	**1.1**
2	2440	853	**35.0**	392	**16.1**	893	**36.6**	271	**11.1**	31	**1.3**
3	2440	1060	**43.4**	362	**14.8**	755	**30.9**	240	**9.8**	23	**0.9**
4	2440	1176	**48.2**	398	**16.3**	624	**25.6**	227	**9.3**	15	**0.6**
5 (highest)	2440	1373	**56.3**	366	**15.0**	462	**18.9**	219	**9.0**	20	**0.8**

* Significant at p < 0.05

** "Other" includes divorced, widowed, separated and unknown

Table 3 shows the smoking prevalence rates and odds ratio inequalities across age groups according to education. An inverse educational gradient was found with statistical significance for all age groups, with the ages 21-30 years old as the only exception. The education inequalities were larger in all age group, and greatest in age 21-30 years old (OR = 5.88, 95%CI = 4.33-7.99), with the prevalence of smoking higher among the lower educated compared to the higher education (OR = 3.26, 95%CI = 2.84-3.75). Odds ratios for the other groups showed an increasing current smoking significantly. The SIIs and the RIIs were negative for all age-groups. The absolute value of SII in 21-30 age-group was highest among that in all age-group categories.

**Table 3:** Smoking prevalence and inequalities in smoking by education level in each age group among males more than 21 years old, 2006 (n = 11,688)

Age-group	Smoking prevalence (in percentage)				Odds ratio**	SII/RII***
			
	Low Education	Middle Education	High Education	Total	(95% CI)	p-value
21-30	57.1	47.2	18.5	40.9	5.88*	-54.3/-1.33
					4.3-8.0	p = 0.18
31-40	54.4	45.8	25.6	46.0	3.47*	-36.7/-0.80
					2.8-4.4	p = 0.24
41-50	52.6	41.2	24.8	44.8	3.37*	-39.3/-0.88
					2.7-4.2	p = 0.16
51-60	47.3	27.4	18.8	40.1	3.88*	-47.2*/-1.18*
					2.8-5.3	p = 0.02
> = 61	36.0	17.1	13.5	32.0	3.61*	-39.6*/-1.24*
					2.2-6.1	p < 0.01
All ages	47.5	39.8	21.7	41.2	3.26*	-33.1/-0.80
					2.8-3.8	p = 0.26

* Significant at p < 0.05

** "Odds ratio" represents the ratio of smoking prevalence among low education subjects to that among high education subjects.

*** "SII" denotes the slope index of inequality and RII relative index of inequality. The RII is the ratio of SII to the age-group specific smoking prevalence.

Table 4 shows the adjusted odd ratios of smoking status across age groups according to socio-economic status. The odds ratios were usually higher among the younger than older men. The highest odds ratio among males aged 21 to 30 year (OR = 2.66, 95%CI = 2.27-3.12) was shown as compared to the males age more than 61 year. The strongest association was found between current smoker and education. The males with lower education were much likely to be current smoker (OR = 3.15, 95%CI = 2.74-3.62) as compared to the males with higher education. With respect to the geographic regions, the males in South region were more likely to be current smoker (OR = 1.30, 95%CI = 1.14-1.48) than the males in Northeast region. The males in Central, Bangkok, and North regions were less likely to be current smoker (OR = 0.83, 95%CI = 0.75-0.92; OR = 0.79, 95%CI = 0.65-0.95; OR = 0.73, 95%CI = 0.66-0.81) than the males in Northeast region. Single and married males were less likely to be current smoker (OR = 0.69 and 0.76, p < 0.05, respectively) than the meals with others marital status. The odds ratio of current smoker compared with the richest quintile was highest among the poorest quintile and the odds ratio declined according to the household income quintile increased (OR = 1.91, 1.55, 1.23, and 1.03, p < 0.05, respective for quintile 1, 2, 3, and 4) compared with the richest quintile 5.

**Table 4:** Cigarette smoking and other socioeconomic variables in a logistic regression analysis among men 21 years of age or older, 2006 (n = 11,688)

Characteristics	Current smokers		
	
	OR	95% CI	
		
		Lower	Upper
**Ages**			
21-30	2.66*	2.27	3.12
31-40	2.60*	2.28	2.95
41-50	2.37*	2.1	2.68
51-60	1.76*	1.55	2.01
61 and older	Reference		
**Education**			
Lower Education	3.15*	2.74	3.62
Middle Education	2.18*	1.91	2.5
High Education	Reference		
**Region**			
Bangkok	0.79*	0.65	0.95
Central	0.83*	0.75	0.92
North	0.73*	0.66	0.81
Northeast	Reference		
South	1.30*	1.14	1.48
**Marital status**			
Single	0.69*	0.57	0.82
Married	0.76*	0.66	0.88
Others marital status	Reference		
**Household income quintile**			
1 (lowest)	1.91*	1.66	2.2
2	1.55*	1.35	1.77
3	1.23*	1.08	1.4
4	1.03	0.9	1.17
5 (highest)	Reference		

* Significant at p < 0.05

**Age more than 61 years old, High education, Northeast region, marital status of "Other", and Quintile 5 as reference groups.

## Discussion

The results showed that 41.5% of adult Thai male population were smokers. A number of socio-demographic factors were associated with smoking status: younger age, lower income, lower education, residents of South region, and the other marital status. Observed social gradients in smoking are steeper in younger age group than in older age group.

Inverse associations between smoking and socioeconomic status are addressed earlier studies carried out in developed countries [2-5,7]. Tobacco use is now more prevalent among low education, manual occupation, and low income. Studies in developing countries [13-18] found a social gradient similar to that in Western countries. However, accumulated effects of multiple factors, such as education and age groups are not addressed in studies with limited number of subjects. Evidence to address particular groups with high smoking prevalence helps identify most vulnerable populations that anti-smoking programs should be provided with priorities.

Success of tobacco control programs was reported on the basis of analysis of policies in the USA, Canada, Sweden, UK and Australia [21]. Their measures included increasing tobacco taxation, limits on advertising and sponsorship, restrictions on smoking in public places, provision of nicotine-replacement therapy, intensive counseling for smoking cessation, and prohibitions of sales to children and health education campaign and have shown a number of successful achievements [22-24]. In Scotland [25], the number of hospitalizations due to acute coronary syndrome declined after the implementation of smoke-free legislation. Price increase has shown to be particularly influential to low socioeconomic class. A review of policies aimed at reducing inequalities in smoking, 10% price increase reduces smoking consumption by about 4% in high-income countries and 8% in low-income countries [26].

We found that smoking prevalence in Thailand were particularly high among younger adults from low-socioeconomic class. The rate of smoking prevalence among low and high education groups among 21-30 age group was higher than that among other age groups. Such a greater difference of smoking prevalence by educational levels would result in greater inequal distribution of health risks in later life by social class.

Low smoking prevalence in Thailand in high education group was remarkable. Reduction of number of people of few educational histories will also contribute to reduce smoking prevalence. Therefore general scaling up of education definitely contribute to reduce smoking prevalence and increase healthy populations. General improvement of socioeconomic status of the society will lead to healthy populations.

Roll-your-own cigarettes are not highly taxed and are sold at affordable price. Such cigarettes of affordable price are accessible for people in low income classes and young adults. Free nicotine-replacement therapies are not available in this country. High pricing policies for both manufactured and non-manufactured cigarettes would be effective to reduce smoking among young and low socio economic status in Thailand. To plan and implement evidence-based anti-smoking policies, there are needs for research on the impact of smoking control policies on reduction of smoking prevalence in low socioeconomic classes.

Our finding suggests that the smoking epidemic still exists in Thailand. Socioeconomic inequalities in smoking, particularly among younger generation are concerned. It is recommended that for better and more effective tobacco control policy should be formulated specifically for the younger age group with lower levels of education. Health education on harmful consequences of smoking and promotion of non-smoking habits should be further emphasized at school education. Improving the educational status of population in developing countries should be able to help control epidemic of smoking and related health inequalities. By considering smoking habits in Thailand, anti-smoking policy measures should be further developed for roll-your-own cigarette. Pricing policies and reductions in the physical availability of tobacco products in neighborhoods should be considered to reduce tobacco consumption

## Conclusions

The inverse associations between smoking prevalence and socioeconomic status among the Thai adult male population were consistently confirmed. The social gradient in smoking was greater among young adult males than that among older adult males. The results suggest the existence of hidden age and social class, young adults coming from low-socioeconomic class, left behind from the decline of smoking in general population.

## Competing interests

The authors declare that they have no competing interests.

## Authors' contributions

SM and KN conceived, designed the study, created database for analysis, conducted statistical analysis and interpreted the results, drafted and completed the manuscript. MK and AM were involved in data analysis and interpretation of the results. TS contributed to acquire dataset for analysis. KS and TT made intellectual input to the draft. All authors read and approved the final draft.
